# Supplementary material for: Targeting the TLK1-MK5 Axis Suppresses Prostate Cancer Metastasis
Source: Cancers (Basel). 2025 Mar 31;17(7):1187. doi: 10.3390/cancers17071187 (PMC11988051; doi:10.3390/cancers17071187)

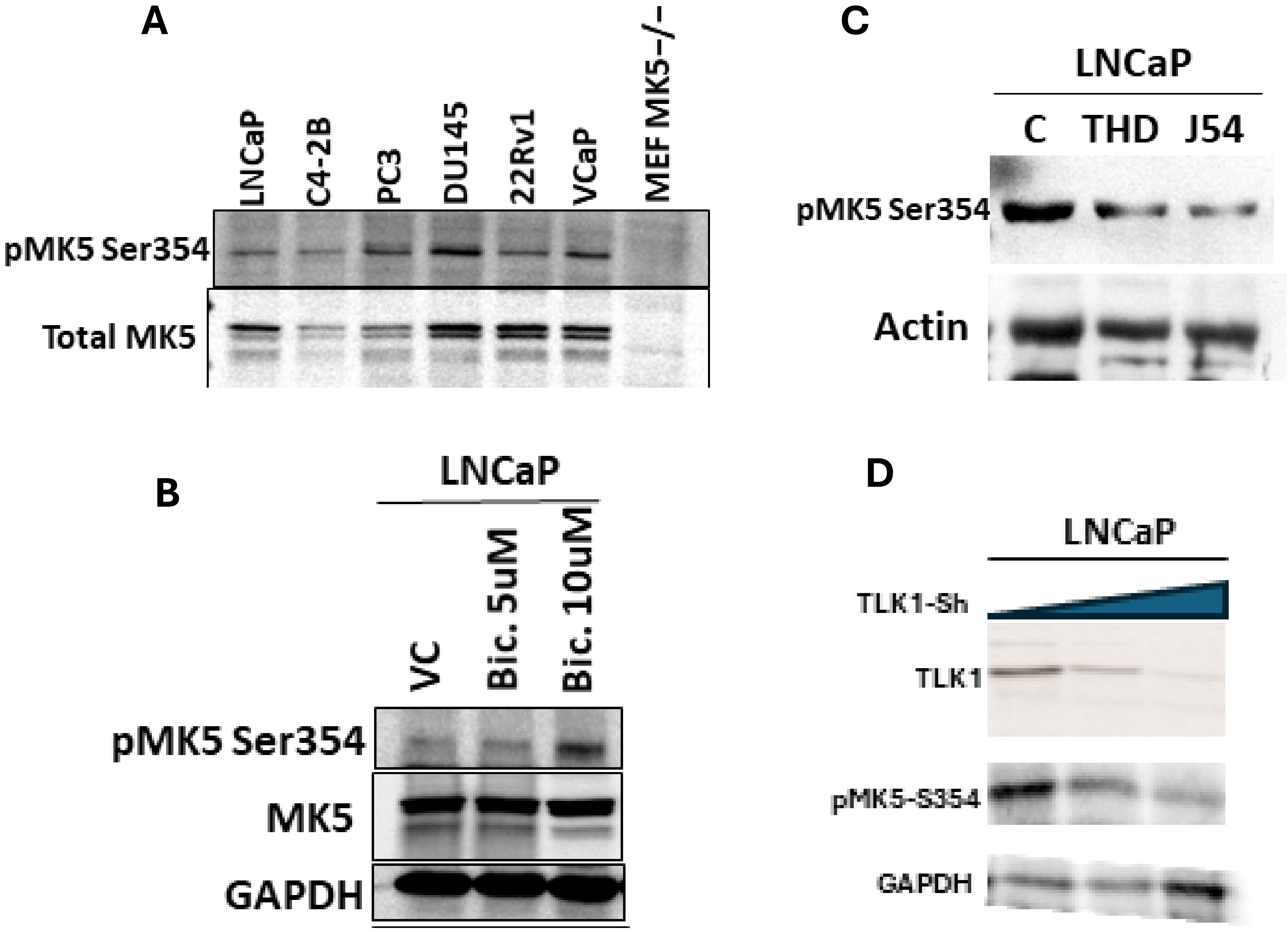

Figure S1. Treatment with ARSI (BIC) increases pM5-S354 and TLK1 inhibition reduces it. A) pMK5, total MK5levels in different Pca cell lines. **B)** pMK5 induction in LNCaP cells after treatment with 2 concentrations of BIC compared to vehicle control (VC). **C)** pMK5 is reduced after treatment with the TLK inhbitors THD or J54. **D)** shRNA-mediated knockdown of TLK1 results in parallel dose-dependent depletion of pMK5.

Figure S2. Effect of J54 treatment on pMK5-S354 isolated from lung tumor nodules (J) from 2 mice, in comparison to untreated controls (T). Th blot was sequentially probed also for TLK1 and HSP70

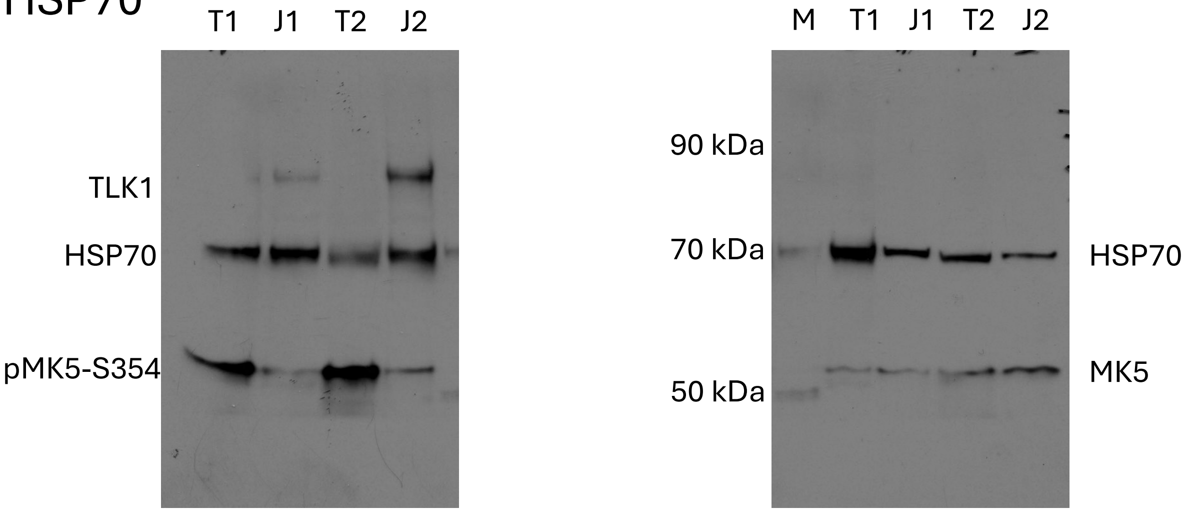

Supplement: Supplementary file 1 [file cancers-17-01187-s001.zip › cancers-3531639-supplementary.pdf]
